# Differential responses of body growth to artificial warming between parasitoids and hosts and the consequences for plant seed damage

**DOI:** 10.1038/s41598-017-15453-y

**Published:** 2017-11-13

**Authors:** Xinqiang Xi, Yangheshan Yang, Xiaocheng Yang, Sören Nylin, Nico Eisenhauer, Shucun Sun

**Affiliations:** 1 Department of Ecology, School of Life Science, Nanjing University, 163 Xianlin Avenue, Nanjing, 210023 China; 2 College of Materials and Chemistry & Chemical Engineering, Chengdu University of Technology, East 3 Road ErXian Bridge ChengHua District, Chengdu, 610059 China; 3 Department of Zoology, Stockholm University, SE-106 91 Stockholm, Sweden; 4 German Centre for Integrative Biodiversity Research (iDiv) Halle-Jena-Leipzig, Deutscher Platz 5e, 04103 Leipzig, Germany; 5 Institute of Biology, Leipzig University, Deutscher Platz 5e, 04103 Leipzig, Germany; 6 Center for Ecological Studies, Chengdu Institute of Biology, Chinese Academy of Sciences, 9 Section 4, Renminnan Rd, Chengdu, 610041 China

## Abstract

Temperature increase may disrupt trophic interactions by differentially changing body growth of the species involved. In this study, we tested whether the response of body growth to artificial warming (~2.2 °C) of a solitary koinobiont endo-parasitoid wasp (*Pteromalus albipennis*, Hymenoptera: Pteromalidae) differed from its main host tephritid fly (*Tephritis femoralis*, Diptera: Tephritidae; pre-dispersal seed predator), and whether the plant seed damage caused by wasp-parasitized and unparasitized maggots (larval flies) were altered by warming. In contrast to the significant and season-dependent effects of warming on body growth of the host tephritid fly reported in one of our previous studies, the effect of artificial warming on body growth was non-significant on the studied wasp. Moreover, the warming effect on seed damage due to unparasitized maggots was significant and varied with season, but the damage by parasitized maggots was not altered by warming. Distinct responses of body growth to warming between parasitoids studied here and hosts assessed in a previous study indicate that temperature increase may differentially affect life history traits of animals along food chains, which is likely to affect trophic interactions.

## Introduction

Climate change may significantly alter species interactions, species diversity, and ecosystem functioning^1,2^. Trophic interactions, which are critical in regulating species population dynamics, community assembly, and flux of material and energy in ecosystems^3,4^, have widely been observed to be sensitive to temperature increase^5,6^. This is because species at different trophic levels often vary considerably in physiological and behavioral responses to environmental change^7–11^. However, the mechanisms underlying warming-induced shifts in trophic interactions and the consequences for the fitness of species involved have not been fully understood^12^. Exploring the mechanisms is particularly important for food chains involving plants, herbivores, and parasitoids or pathogens, where outbreaks of notorious pests and pathogens are likely to occur if trophic relationships are decoupled by climate change^13–16^.

Typically, climate change can shift trophic interactions by causing temporal or spatial mismatches between species^9–11,17^. For example, phenological synchronies are often disrupted^18^, and inconsistent shifts in species distributions and habitat domains are often induced by temperature increase between parasites and host species^19^, increasing the frequency of outbreaks of insect pests or pathogens^15,20^. Trophic interactions can be altered by climate change, for instance, through differentially affecting body growth of the involved species^21^, which may also affect the fitness of species at lower or higher trophic levels of the interacting species. Despite numerous examples demonstrating these mismatches, in most cases temporal or spatial overlaps remain between consumer and resource species^14,22,23^.

Temperature increase has been demonstrated to change species’ traits, for example, body size^24–26^. However, if changes in body size differ between parasitoids and their host species, the trophic relationship within these food chains could be disrupted as larger parasitoids are often associated to larger herbivore hosts^27^. Moreover, body size is often positively correlated with lifetime reproductive success^28–31^ and mobility^32^. Thus, differential body-size responses of species in a food chain could shift population dynamics of species at different trophic levels^33,34^ with potential consequences for ecological communities^14,35,36^.

Temperature increase is known to reduce the body size of both vertebrate and invertebrate animals^24^, but not all the species at different trophic levels decrease body size to a similar extent^33^, and some species may even increase body size under experimental warming^37,38^. Temperature increase may shift energy budgets, growth and development period, and food availability to alter species body size^24,37,39^. Predators and their prey, or parasitoids and their hosts, often differ in body size-volume ratio, which is intimately related to energy budgets, foraging strategies as well as life-history traits^40^. It is therefore expected that species at different trophic levels may have different body-size responses to temperature increase^12,41^. For example, temperature increase delayed the emergence and increased the body size of herbivorous caterpillars (*Euphydryas aurinia*) but not their parasitoid wasps (*Cotesia bignellii*) in a field experiment^42^. In addition, artificial warming increased the body size of herbivorous insect species (mostly *Persectania aversa* and *Tmetolophota unica*) but not that of their parasitoids in a grassland in New Zealand^43^. However, empirical evidence is still sparse for inconsistent warming-induced shifts in life history traits along food chains, and the associated consequences for trophic interactions have rarely been explored^21^.

In this study, we used a food chain of parasitoid wasp-tephritid fly-Asteraceae plant to test the possible effect of temperature increase on trophic interactions in an alpine meadow in the eastern part of the Tibetan Plateau. Parasitoid-herbivore-host plant interactions are among the most important trophic interactions in nature, and more than half of the multicellular species were found to be involved in such parasitic food chains^44,45^. For the studied food chain, our previous work shows that the seeds within the capitula of Asteraceae species are heavily consumed by pre-dispersal seed predators (tephritid flies), whose larval maggots can be parasitized by koinobiont wasp species^46^. We have also found that the parasitoid wasps may modify the behavior of their hosts, allowing the seed consumption rate of the parasitized maggots to be greater than that of unparasitized ones^46^. In particular, our previous work has shown that artificial warming could significantly reduce the body size of the tephritid flies in the early season, but increase their body size in late season^38^. In the current study, we further examined the warming effect on body growth (as reflected by adult body mass and development time) of the wasps to test whether artificial warming induce a similar body-size response in flies and wasps. Moreover, we examined seed damage caused by parasitized and unparasitized maggots under warmed and ambient conditions, aiming to test whether altered trophic interactions cascade to affect seed damage in the plants. Additionally, both the fly and wasp species are multivoltine, having two or more generations per year and having different host plant species at different periods of the growing season in the study area. Thus, we also determined if the warming effect on the body growth of parasitoid wasps as well as plant seed damage induced by parasitized and unparasitized maggots depend on the season/host plant species.

## Materials and Methods

### Study site

This study is part of a series of artificial warming experiments in the field aimed to test for the effect of climate change on the “Asteraceae plants-tephritid flies-parasitoid wasps” food chain^38^. The study was conducted in Hongyuan County, Sichuan province, China (32 °48′N, 102 °33′E), in the eastern part of the Tibetan Plateau. The altitude is ca. 3,500 m above sea level and the climate is characterized by a short and cool summer and a long and cold winter. Mean annual temperature is 0.9 °C, with the maximum monthly average temperature being 10.9 °C (July), and the minimum monthly average temperature being −10.3 °C (January)^47^. Meteorological data from 1961 to 2012 collected by the local climate station showed that the mean annual temperature has increased at a rate of 0.29 °C per decade^48,49^.

Alpine meadow is the major vegetation type in the study area. Plant coverage mostly exceeds 90% of the soil surface in the meadow. Sedges like *Kobresia setchwanensis* and *Blysmus sinocompressus* dominate lowland and high soil moisture communities, while forbs like *Potentilla anserina, Saussurea nigrescens*, and *Anemone trullifolia* var*. linearis* are dominant species in communities with relatively low soil moisture.

### Study species

Three Asteraceae plant species, one shared parasitic pre-dispersal seed predator and its parasitoid wasp (Hymenoptera) were included in this study. All the three Asteraceae species are common in the study area in different seasons, and they are dominant host plants for the studied tephritid fly in their respective flowering season. *Cremanthodium brunneopilosum* flowers in late May to mid-June, and plants used in the present experiment were on average 52.19 ± 2.69 cm (mean ± se) high, having 4.51 ± 0.3 capitula per plant and 170.6 ± 11.17 florets per capitulum (*N = *30). *Saussurea nigrescens* flowers in mid-June to late August, and plants used in the present experiment were on average 26.76 ± 0.96 cm high, having 3.35 ± 0.46 capitula per plant and 38.2 ± 2.5 florets per capitulum (*N* = 30). *Saussurea stella* flowers in mid-August to mid-September, and plants used in the present experiment were less than 5 cm high, having 4–11 capitula per plant and 18.1 ± 0.45 florets per capitulum (*N = *30)^46^. The fruiting periods of all these focal host species were longer than the development time of both unparasitized and parasitized maggots. Flowering phenologies of these three plants are shown in Fig. S1.

The seed predator is a multivoltine tephritid fly species (*Tephritis femoralis* Chen, 1938; Diptera: Tephritidae)^46^, whose larvae consume developing seeds of Asteraceae plant species and whose larvae can be parasitized by parasitoid wasps. After overwintering in the soil, adult flies emerge and mate in spring, the mated females oviposit into the unflowered capitula of Asteraceae host plant species. Larval maggots feed on the immature seeds and sometimes on parts of receptacles of the host plants; they would not move out of plant capitula before developing into adults. The fly species usually has two to three generations per year in the studied meadow. The host species are *C*. *brunneopilosum* for the first generation, *S*. *nigrescens* for the second and sometimes the third generation (because of a long flowering period), and third generation of the tephritid flies also hosted by *S*. *stella*.

*T*. *femoralis* maggots are often parasitized by the parasitoid wasp *Pteromalus albipennis*, while they are growing in the capitula. *P*. *albipennis* is a solitary koinobiont endo-parasitoid, which allows parasitized maggots to continue consuming seeds, and all of the parasitized maggots are killed after they finished pupation. *P*. *albipennis* is also a multivoltine insect having two to three generations per year, matching the population dynamics of the fly species. In addition, *P*. *albipennis* is a generalist parasitoid wasp that can be hosted by several tephritid flies in the genera *Tephritis* and *Campiglossa*. However, *T*. *femoralis* is the main hosts for *P*. *albipennis*, accounting for more than 80% of the host flies for *P*. *albipennis* in all three plant species, while *P*. *albipennis* accounts for more than 75% of the wasps that attack larval *T*. *femoralis* in the capitula of studied plant species^50^.

### The warming experiment

We conducted an artificial warming experiment to test for the effect of temperature increase on body growth of the tephritid fly and the parasitoid wasp and also on plant seed damage due to (wasp) parasitized and un-parasitized maggots in a manipulated tritrophic food chain. The body size response to experimental warming in the tephritid fly has been reported in one of our previous studies^49^. In this paper, we focused on the warming effect on body growth of parasitoids as well as on plant seed damage caused by unparasitized and parasitized maggots.

Our experiment had 12 treatments in total, i.e. temperature (warming vs. ambient) × insects (fly vs. wasp) × plants (*C*. *brunneopilosum*, *S*. *nigrescens*, and *S*. *stella*), with each treatment having 15 replicates. We inserted one *P*. *albipennis*-parasitized or unparasitized maggot (ca. 2 mm in length) into one randomly chosen plant capitulum. Then, the capitula bearing maggots were bagged by fine meshed nylon screens to prevent additional parasitism and to prevent the emerged flies or wasps from escaping. Moreover, they were cultivated under both warmed and ambient conditions. These procedures were replicated for all the three host plant species that flowered in early, intermediate, and late growing season, respectively (see also Fig. S1 for details).

The artificial warming was achieved by infrared heaters (1600W, Heraeus Company, Germany). The heaters were hung at a height of 1.3 m above the capitula to warm the maggots within the plant capitula in the warmed treatment. Similar dummy heaters were also hung above plants in the ambient treatment to control for potential side effects of the construction. The infrared heaters used in this study were 1 m in length and can evenly heat the 2 m × 2 m plots ~1.5 m under the heater^38^, so that the plants distributed within plots were evenly heated^38^. Infrared heaters were turned on as soon as the maggots were inserted into the capitula and were turned off after all of the flies or wasps had emerged out of the capitula. Records from Hobo U23-003 temperature data loggers (Onset Company, USA) showed that temperature at the height of flowerhead was increased by 2.15 °C, 2.07 °C, and 2.32 °C during the experiments with the early, intermediate, and late flowering plant species, respectively (Fig. S1).

Experimental plants were transplanted from a natural meadow in the study area at the end of September, 2013. About 150 middle-sized mature plant individuals and the associated soil were dug out for each host plant species and immediately moved to plastic pots. All these plants were cultivated in our research station, and they were all artificially pollinated and irrigated regularly.

Experimental maggots were derived according to the same methods as done in our previous studies^38,46,50^. We grew host plants in 2 m × 2 m × 2 m cages covered by a 0.18 × 0.18 mm meshed nylon screen, from which insects had been removed beforehand. Then, newly mated female *T. femoralis* were released into the cages for three days to oviposit into the capitula, after which they were driven out of the box. On the 9th day after the release of adult flies into the boxes, we opened the capitula carefully and collected the maggots. Detailed procedures are shown in one of our previous studies^38^.

*P*. *albipennis*-parasitized maggots were obtained by parasitizing the collected maggots with newly mated female parasitoid wasps. We collected adult wasps directly from the field two days before collecting the unparasitized maggots. The collected adult wasps were released into a 0.8 × 0.7 × 0.5 m (length × width × height) plastic box, in which they were allowed to mate freely. Then, mated females were put into glass bottles (10 cm in both diameter and depth), where a fly maggot had been placed. A maggot was considered to be parasitized if the female had inserted its ovipositor into the maggot body for at least 10 s. Each female parasitoid was allowed to parasitize one maggot only.

We checked whether adult insects emerged from the bagged capitula every day. Because adults always crawled out of the plant capitula shortly after their emergence, the date we observed the adult wasps was exactly the date of the emergence. We therefore recorded the development time as the days from the date of the start of the experiment to the date of adult emergence. The emerged adults were immediately put into 10 ml plastic centrifuge tubes, which were placed in a 0 °C refrigerator for 2 h, and subsequently the fresh mass was measured for each adult using an electronic balance (Sartorious Company, precision 0.0001 g)^42,43^. Later, the bagged capitula were cut from the host plants, and the total number of seeds (indicated by the number of flower tubes) and intact seeds (without holes in the seed surface) were recorded. The number of damaged seeds was calculated by the subtracting the number of intact seeds from the total number of seeds as described in^42^. Then, intact seeds were dried at 65 °C for 48 h, and their mass was measured. Individual seed mass was calculated by dividing intact seed mass by the number of intact seeds.

### Data analysis

Three-way analyses of variance (ANOVAs) were employed to determine the effect of warming (ambient vs. warmed), host plant (early, intermediate, and late flowering species), and sex (female vs. male) on the development time and adult fresh mass of the wasps. Then, three-way ANOVAs were employed to determine the effect of warming, host plant, and sex on the number of damaged seeds (per capitulum) in three host plants induced by unparasitized and *P*. *albipennis*-parasitized maggots, respectively. Two-way ANOVAs were employed to test whether warming and parasitism (parasitized vs. unparasitized maggots) significantly changed the total number of seeds per capitulum and individual seed size of each host plant species, respectively. Tukey’s HSD *post hoc* tests were used to determine the differences among treatments once a significant effect was detected. In addition, general linear models were employed to determine the relationship between seed damage and fresh body mass of the adult fly or wasp emerged from the capitula. All data analyses were conducted using R 3.1.0^51^. As explained above, the readers should note that season/insect generation effects could not be separated from plant species-specific effects, and thus, the host plant effect serves as a proxy for the season effect or generation effect.

### Data availability

The datasets generated during and/or analysed during the current study are available from the corresponding author on reasonable request.

## Results

### Warming effects on insect body growth

**Figure 1:**
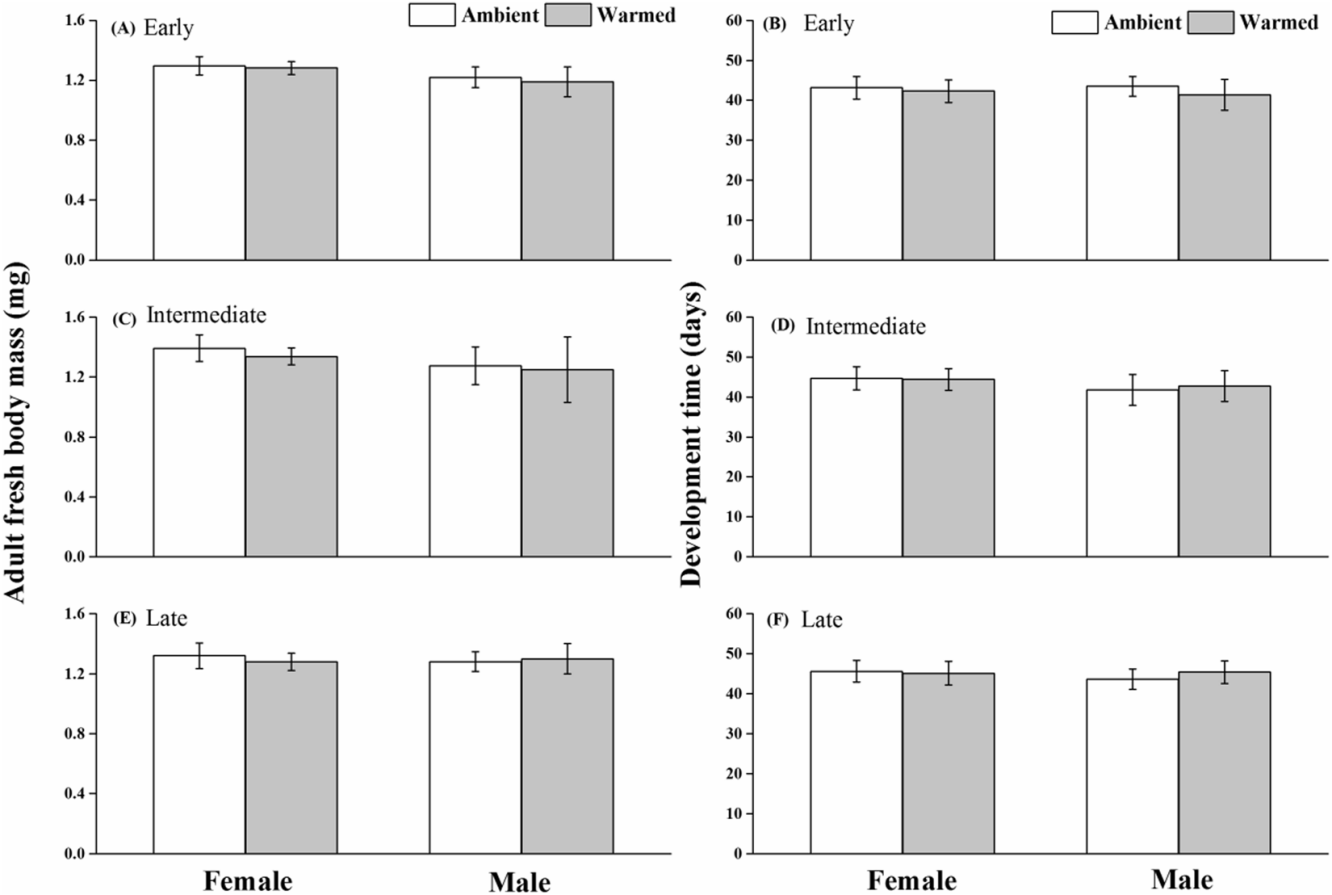
Development time and adult fresh mass of the parasitoid wasp *Pteromalus albipennis* grown in host fly larvae within the plant species *Cremanthodium brunneopilosum*, *Saussurea nigrescens*, and *Saussurea stella* flowering in early (**A**,**B**), intermediate (**C**,**D**), and late (**E**,**F**) growing season and in both ambient and warmed environments, respectively. The different letters above the columns indicate significant differences between treatments as detected by ANOVAs, followed by *post hoc* Tukey’s HSD tests. Error bars denote standard errors.

The warming effect on body growth differed significantly between the parasitoid and the tephritid fly and varied among host plant species/seasons (Table 1; Fig. 1; see also Fig. 1 in^38^). In our previous work, we found that life-history traits of the tephritid fly were plastic and differed significantly among host plant species; artificial warming shifted both development time and adult fresh mass of unparasitized maggots; and the warming effect differed with host plant species^38^. By contrast, here we found that the studied parasitoid had less plastic life-history traits compared to the tephritid fly: the warming effect was non-significant on development time as well as adult fresh mass of the parasitoid wasp in all host plant species (Table 1; Fig. 1).

**Table 1:** ANOVA results showing the effects of warming, plant species, and sex on the development time and adult fresh mass of the parasitoid wasp.

	d.f	Development time			Adult fresh mass		
		SS	F	P	SS	F	P
Warming (W)	1	27.3	1.5	0.23	0.11	2.60	0.11
Plant (P)	2	64.4	1.7	0.19	0.21	2.50	0.09
Sex (S)	1	3.9	0.2	0.65	0.003	0.06	0.80
W*P	2	52.6	1.4	0.25	0.011	0.14	0.87
W*S	1	25.1	1.3	0.25	0.11	0.28	0.61
P*S	2	6.1	0.2	0.85	0.062	0.76	0.47
W*P*S	2	93.9	2.5	0.09	0.032	0.40	0.67
Residuals	70	1303.7			27.8		

d.f = degrees of freedom; SS = Sum of Squares; F = F value; P = P value.

### Warming effect on plant production and seed damage

**Figure 2:**
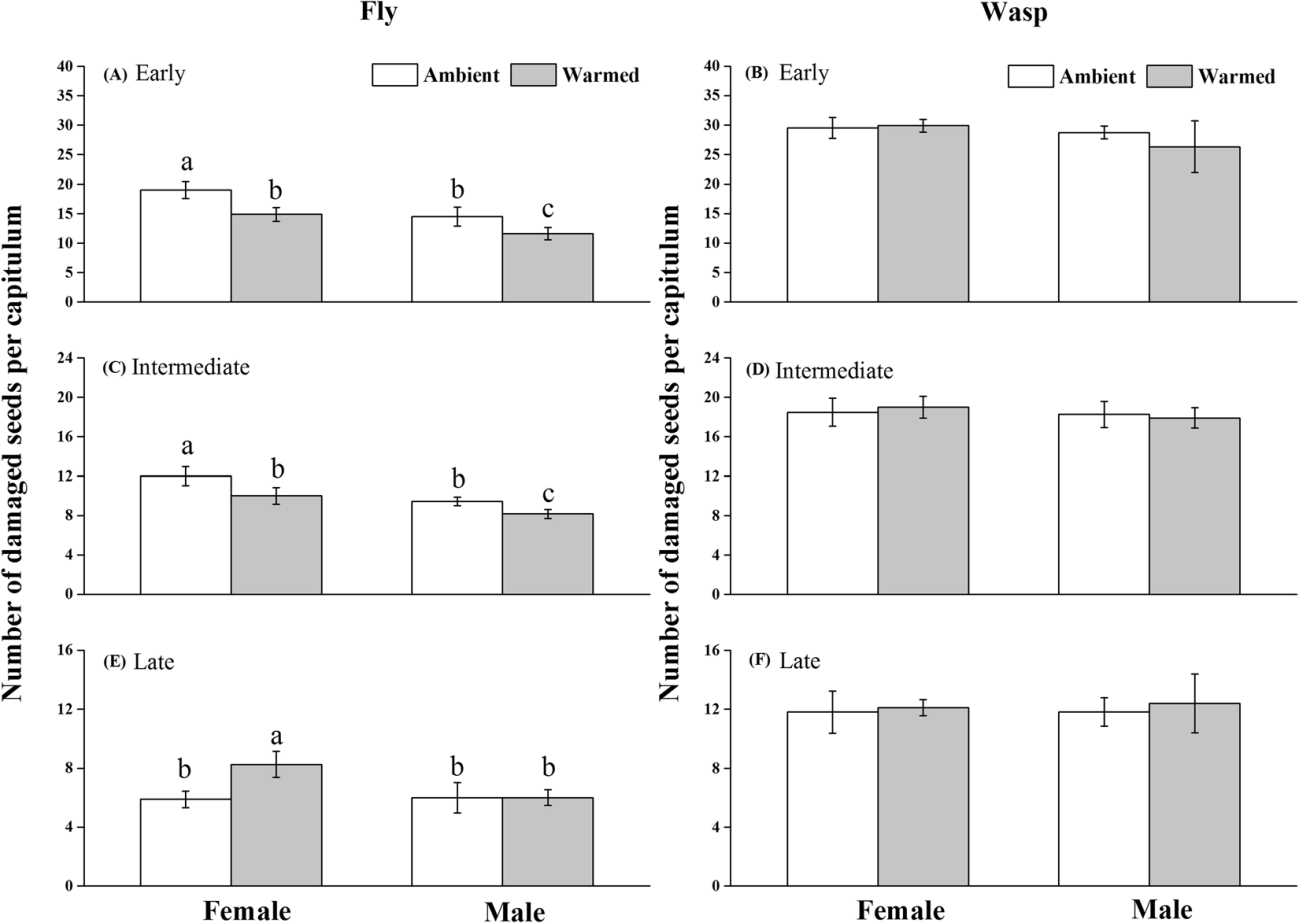
Seed damage (number of damaged seeds per capitulum) in response to feeding of unparatized maggots of a tephritid fly (fly) and maggots parasitized by wasps (wasp) that were grown in *Cremanthodium brunneopilosum*, *Saussurea nigrescens*, and *Saussurea stella* plants flowering in early (**A**), intermediate (**B**), and late (**C**) growing seasons, respectively, in ambient and warmed plots. The different letters above the columns indicate significant differences among means as detected by ANOVAs, followed by *post doc* Tukey’s HSD test.

Artificial warming did not change the total number of seeds per capitulum (Fig. S2), and it did not change individual seed mass in all the three plant species (Fig. S3). However, warming significantly affected seed consumption by unparasitized maggots living in all three host plant species (Table 2). Specifically, warming decreased seed consumption by about 21% in the early season and by 17% in the intermediate season, but it increased seed consumption by 40% in the late flowering plant species (Fig. 2). In contrast, the warming effect on seed damage induced by wasp- parasitized maggots was not significant (Table 1; Fig. 2).

**Table 2:** ANOVA results showing the effect of warming, plant species, and sex on the seed damage caused by tephritid fly (*Tephritis femoralis*) and wasp (*Pteromalus albipennis*) parasitized maggots.

	Tephritid fly				Parasitoid wasp			
	d.f	SS	F	P	d.f	SS	F	P
Warming (W)	1	62.5	8.4	**0.005**	1	9.4	0.71	0.40
Plant (P)	2	1152.6	77.8	**<0.001**	2	4543	172	**<0.001**
Sex (S)	1	100.8	13.6	**<0.001**	1	8.2	0.62	0.43
W*P	2	105.0	7.1	**0.001**	2	8.6	0.32	0.72
W*S	1	0.04	0.01	0.94	1	2.4	0.18	0.67
P*S	2	32.3	2.2	0.12	2	19.0	0.72	0.49
W*P*S	2	13.6	0.92	0.40	2	30.3	1.15	0.32
Residuals	78	577.5			70	923.9		

d.f = degrees of freedom; SS = Sum of Squares; F = F value; P = P value.

**Figure 3:**
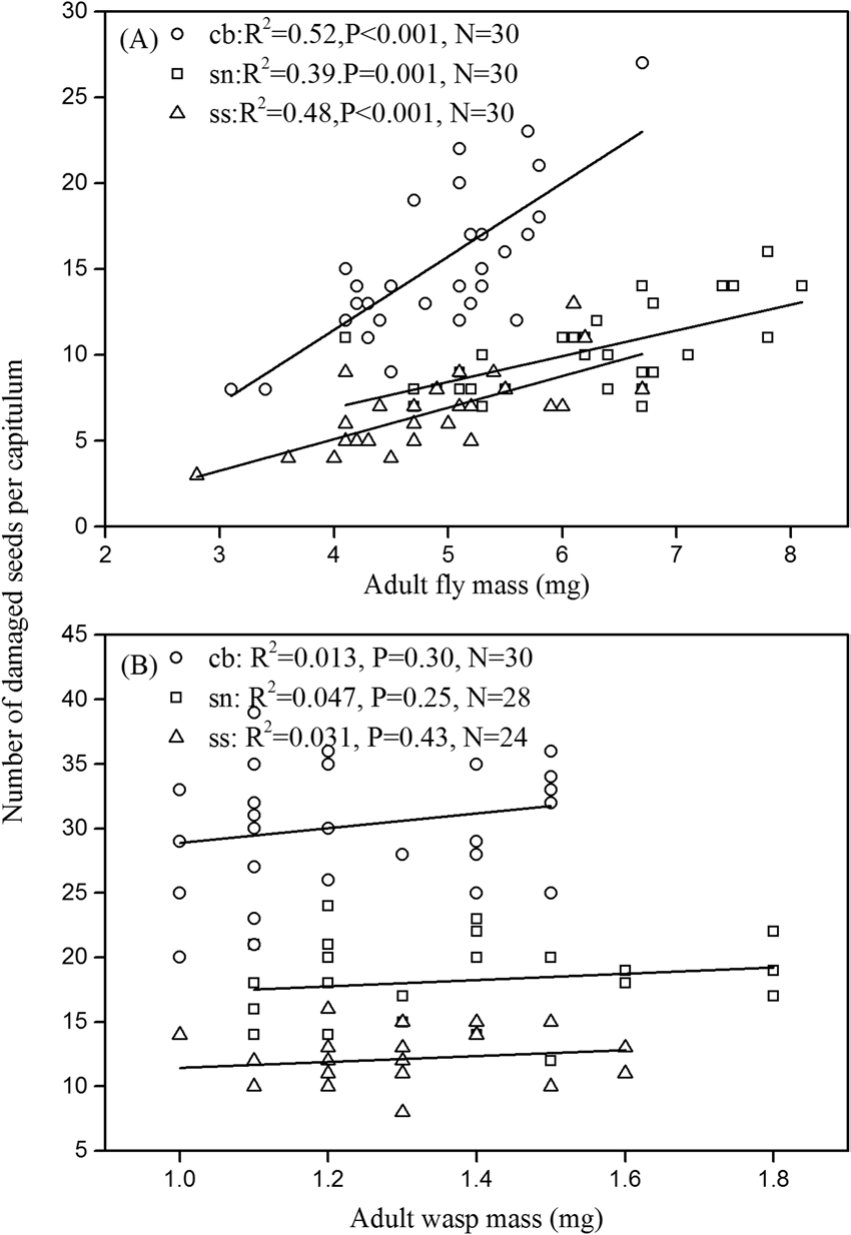
Regressions between adult body mass (of flies and wasps) and number of damaged seeds for (**A**) unparasitized and (**B**) parasitized maggots, both of which were grown in three Asteraceae host plant species (cb, *Cremanthodium brunneopilosum*; sn, *Saussurea nigrescens*; ss, *Saussurea stella*). r^2^, N, and P values are provided.

In addition, seed damage caused by unparasitized maggots increased with body mass of the tephritid fly (Fig. 3A), whereas there was no significant relationship between seed damage caused by wasp-parasitized maggots and adult wasp mass (Fig. 3B).

## Discussion

Contrary to the significant effect of warming on the life-history traits of host tephritid flies assessed in our previous study^38^, we here show that experimental warming has a non-significant effect on both the development time and body mass of the studied parasitoid wasp species. Experimental warming consistently altered the seed damage caused by unparasitized maggots, but not seed damage caused by parasitized maggots because of the positive relationship between seed damage and body growth. Importantly, the warming effects on insect body growth and seed damage varied with plant species/season. These findings may have implications for predictions about the trophic interactions between parasitoids and hosts and their impact on plant regeneration in a warming world.

In contrast to the host tephritid fly species (unparasitized maggots), the wasp species (parasitized maggots) was insensitive to warming: the warming effect was consistently non-significant for the three generations in the three host plant species over the growing season. Such insensitivity is consistent with results of previous controlled, semi-natural field experiments^42,43^, in which parasitoids were found to show no significant response in body growth to experimental warming. However, our finding is in contrast to the results of a growth chamber experiment with constant temperature setting, in which the growth and development rates of a Braconidae wasp (*Apanteles bignellii*) were found to be temperature-dependent^52^. The discrepancy between field experiments and chamber experiments may be at least partly attributed to the difference in parasitoid performance between constant versus fluctuating temperature conditions in the lab and in the field, respectively^42,43^. Studies have shown that temperature fluctuations affect the life history responses of insects to environmental change^53^.

The differential responses to warming between host tephritid flies and parasitoid wasps may have physiological and evolutionary reasons^42^. On the one hand, koinobiont parasitoids can physiologically manipulate the growth and development of their parasitized hosts to obtain sufficient amounts of resources for their growth and development, mostly by prolonging the larval stage of the parasitized hosts^35,54,55^. The developing parasitoid larvae often do not consume vital organs of their herbivore hosts until they reach a certain size^56^. This manipulative ability may allow parasitoids to be physiologically less responsive to environmental change than their hosts. On the other hand, phenological matching should not be evolutionally favored for herbivorous host-parasitoid wasp relationships^57,58^, because highly synchronous phenologies would lead to high mortality rate, severe reductions in resource populations, and large population oscillations^59^. In our case, the parasitoid wasps are extremely efficient in killing their tephritid fly hosts (100% in the present experiment). Considering that the parasitism rate is usually high, there would be large population fluctuations for both wasp and fly species if a close phenological match persisted for generations. Indeed, experimental warming shortened the development time of the host fly by more than 4 days in early and intermediate seasons^38^. This would narrow the window vulnerable for wasp parasitism, as suggested by the “slow growth-high mortality” hypothesis for larval animals^47,60,61^. Nevertheless, the timing of emergence normally varies much among individual flies and wasps in the field, such that warming-induced phenological changes in the tephritid flies may not necessarily lead to the uncoupling of the fly-parasitoid relationship.

The artificial warming not only differentially affected the body growth of tephritid flies and parasitoid wasps, but also differentially altered the seed damage of host plant species. Because the warming effect was significant on the body growth of the unparasitized maggots but not on parasitized maggots, and because body growth rates were positively associated with seed plant damage, it is not surprising that the warming effect was significant for seed damage caused by unparasitized maggots but not parasitized maggots. However, the warming effect on seed damage caused by unparasitized maggots was host plant species-dependent, as a result of the plant species-specific response of body growth to warming in the unparasitized maggots. The artificial warming decreased the seed damage in the early and intermediate flowering species but increased the seed damage in the late flowering species. It can thus be speculated that these early and intermediate flowering species would suffer less seed damage due to tephritid flies and hence increase their community dominance, whereas the relative abundance of the late flowering species would decrease under future warmed conditions, because these host plant species in the studied tri-trophic food chain rely heavily on seed production for regeneration.

It is worthwhile to note that we did not take into account the warming effect on the abundance of the studied plants and insects, which is likely to affect the interactions between wasps and host flies as well as of flies on host plants. Furthermore, the artificial warming treatment was not carried out for a whole growing season. This may limit our capacity to accurately predict the response of trophic interactions to warming as well as their consequences in natural ecosystems. Large and closed experimental facilities are needed to address this limitation and to more comprehensively appreciate the responses of plant-animal interactions to increasing temperatures. Moreover, as we were unable to separate plant species identity from season effects in the present study, more experiments should be carried out to explore the underlying mechanisms of differential responses to temperature increase. Nevertheless, the present results show that warming differentially affects the body growth of parasitoids and their host tephritid fly, which altered their interactions as well as plant fitness. Mechanisms underlying the effect of temperature increase on trophic interactions may be more common in high-altitude/elevation ecosystems, where species have limited potential to migrate to cooler areas under warming conditions^39^.

## Electronic supplementary material

Supplementary material

## References

[1] Tylianakis JM, Didham RK, Bascompte J, Wardle DA (2008). Global change and species interactions in terrestrial ecosystems. Ecol. Lett..

[2] Suttle KB, Thomsen MA, Power ME (2007). Species interactions reverse grassland responses to changing climate. Science.

[3] Paine RT (1966). Food web complexity and species diversity. Am. Nat..

[4] Estes JA (2011). Trophic downgrading of planet earth. Science.

[5] Both C, Van Asch M, Bijlsma RG, Van Den Burg AB, Visser ME (2009). Climate change and unequal phenological changes across four trophic levels: constraints or adaptations?. J. Anim. Ecol..

[6] van Asch M, Visser ME (2007). Phenology of forest caterpillars and their host trees: the importance of synchrony. Annu. Rev. Entomol..

[7] Harrington R, Woiwod II, Sparks T (1999). Climate change and trophic interactions. Trends Ecol. Evol..

[8] Winder M, Schindler DE (2004). Climate change uncouples trophic interactions in an aquatic ecosystem. Ecology.

[9] Durant JM, Hjermann DØ, Ottersen G, Stenseth NC (2007). Climate and the match or mismatch between predator requirements and resource availability. Clim. Res..

[10] Barton BT, Beckerman AP, Schmitz OJ (2009). Climate warming strengthens indirect interactions in an old-field food web. Ecology.

[11] Liu Y, Reich PB, Li G, Sun S (2011). Shifting phenology and abundance under experimental warming alters trophic relationships and plant reproductive capacity. Ecology.

[12] Dell AI, Pawar S, Savage VM (2014). Temperature dependence of trophic interactions are driven by asymmetry of species responses and foraging strategy. J. Anim. Ecol..

[13] Hance T, van Baaren J, Vernon P, Boivin G (2007). Impact of extreme temperatures on parasitoids in a climate change perspective. Annu. Rev. Entomol..

[14] Jeffs CT, Lewis OT (2013). Effects of climate warming on host-parasitoid interactions. Ecol. Entomol..

[15] Stireman JO (2005). Climatic unpredictability and parasitism of caterpillars: implications of global warming. Proc. Natl. Acad. Sci.USA.

[16] Dong Z, Hou R, Ouyang Z, Zhang R (2013). Tritrophic interaction influenced by warming and tillage: A field study on winter wheat, aphids and parasitoids. Agr Ecosyst Environ.

[17] Vucic-Pestic O, Ehnes RB, Rall BC, Brose U (2011). Warming up the system: higher predator feeding rates but lower energetic efficiencies. Glob. Change Biol..

[18] Evans EW, Carlile NR, Innes MB, Pitigala N (2013). Warm springs reduce parasitism of the cereal leaf beetle through phenological mismatch. J. Appl. Entomol..

[19] Menéndez R, GonzÁLez-MegÍAs A, Lewis OT, Shaw MR, Thomas CD (2008). Escape from natural enemies during climate-driven range expansion: a case study. Ecol. Entomol..

[20] Nelson WA, Bjørnstad ON, Yamanaka T (2013). Recurrent insect outbreaks caused by temperature-driven changes in system stability. Science.

[21] Tylianakis, J. M. & Binzer, A. Effects of global environmental changes on parasitoid–host food webs and biological control. *Biol Contrl* (2013).

[22] Forrest JRK (2016). Complex responses of insect phenology to climate change. Current Opinion in Insect Science.

[23] Teplitsky C, Millien V (2014). Climate warming and Bergmann’s rule through time: is there any evidence?. Evol Appl.

[24] Gardner JL, Peters A, Kearney MR, Joseph L, Heinsohn R (2011). Declining body size: a third universal response to warming?. Trends Ecol. Evol..

[25] Atkinson D (1994). Temperature and organism size-a biological law for ectotherms?. Advan. Ecol. Res..

[26] Atkinson, D. *Ectotherm life-history responses to developmental temperature*. 183–204 (Cambridge University Press, 1996).

[27] Cohen JE, Jonsson T, Müller CB, Godfray HCJ, Savage VM (2005). Body sizes of hosts and parasitoids in individual feeding relationships. Proc. Natl. Acad. Sci. USA.

[28] Bezemer TM, Harvey JA, Mills NJ (2005). Influence of adult nutrition on the relationship between body size and reproductive parameters in a parasitoid wasp. Ecol. Entomol..

[29] Ellers J, Jervis M (2003). Body size and the timing of egg production in parasitoid wasps. Oikos.

[30] Honěk A (1993). Intraspecific Variation in Body Size and Fecundity in Insects: A General Relationship. Oikos.

[31] Peters, R. H. *The Ecological Implications of Body Size*. (Cambridge University Press, 1983).

[32] Elzinga JA, Van Nouhuys S, Van Leeuwen D-J, Biere A (2007). Distribution and colonisation ability of three parasitoids and their herbivorous host in a fragmented landscape. Bas. Appl. Ecol..

[33] Brose U (2012). Climate change in size-structured ecosystems. Phil. Trans. R. Soc. B.

[34] Lurgi M, Lopez BC, Montoya JM (2012). Climate change impacts on body size and food web structure on mountain ecosystems. Proc. Roy. Soc. Lond. B. Bio..

[35] Harvey JA, Molina AC, Bezemer TM, Malcicka M (2015). Convergent development of a parasitoid wasp on three host species with differing mass and growth potential. Entomol. Exp. Appl..

[36] Brose U (2006). Consumer-resource body-size relationships in natural food webs. Ecology.

[37] Bickford, D. P., Sheridan, J. A. & Howard, S. D. Climate change responses: forgetting frogs, ferns and flies? *Trends Ecol Evol* **26**, 553–554; author reply 555–556 (2011). 10.1016/j.tree.2011.06.016 21782274

[38] Xi X, Wu X, Nylin S, Sun S (2016). Body size response to warming: time of the season matters in a tephritid fly. Oikos.

[39] Parmesan C (2006). Ecological and evolutionary responses to recent climate change. Annu. Rev. Ecol. Evol. S..

[40] Raffel TR, Martin LB, Rohr JR (2008). Parasites as predators: unifying natural enemy ecology. Trends Ecol Evol.

[41] Carey, N. & Sigwart, J. D. Size matters: plasticity in metabolic scaling shows body-size may modulate responses to climate change. *Biol*. *Lett*. **10** (2014). 10.1098/rsbl.2014.0408 PMC4155909 25122741

[42] Klapwijk MJ, GrÖBler BC, Ward K, Wheeler D, Lewis OT (2010). Influence of experimental warming and shading on host–parasitoid synchrony. Glob. Change Biol..

[43] de Sassi C, Staniczenko PP, Tylianakis JM (2012). Warming and nitrogen affect size structuring and density dependence in a host-parasitoid food web. Phil Trans Roy Soc B-Biol Sci.

[44] Strong, D. R., Lawton, J. H. & Southwood, S. R. *Insects on plants. Community patterns and mechanisms*. (Blackwell Scientific, 1984).

[45] Hawkins, B. A. *Pattern and process in host-parasitoid interactions*. (Cambridge University Press, 2005).

[46] Xi X, Eisenhauer N, Sun S (2015). Parasitoid wasps indirectly suppress seed production by stimulating consumption rates of their seed-feeding hosts. J. Anim. Ecol..

[47] Zhao J, Yang Y, Xi X, Zhang C, Sun S (2014). Artificial warming facilitates growth but not survival of plateau frog (*Rana kukunoris*) tadpoles in presence of gape-limited predatory beetles. PLoS ONE.

[48] Mu J (2015). Artificial asymmetric warming reduces nectar yield in a Tibetan alpine species of Asteraceae. Ann. Bot..

[49] Xi X, Li D, Peng Y, Eisenhauer N, Sun S (2016). Experimental warming and precipitation interactively modulate the mortality rate and timing of spring emergence of a gallmaking Tephritid fly. Sci. Rep..

[50] Xi X, Yang Y, Yang Y, Segoli M, Sun S (2017). Plant-mediated resource partitioning by coexisting parasitoids. Ecology.

[51] R Development Core Team. R: A language and environment for statistical computing. R Foundation for Statistical Computing, Vienna, Austria. URL http://www.R-project.org/ (Acessed 1th June, 2014).

[52] Porter K (1983). Multivoltinism in *apanteles bignellii* and the influence of weather on synchronisation with its host *euphydryas aurinia*. Entomologia Experimentalis Et Applicata.

[53] Straile D, Kerimoglu O, Peeters F (2015). Trophic mismatch requires seasonal heterogeneity of warming. Ecology.

[54] Pennacchio F, Strand MR (2006). Evolution of developmental strategies in parasitic hymenoptera. Annu. Rev. Entomol..

[55] Vinson SB, Iwantsch G (1980). Host suitability for insect parasitoids. Annu. Rev. Entomol..

[56] Jervis MA, Ellers J, Harvey JA (2008). Resource acquisition, allocation, and utilization in parasitoid reproductive strategies. Annu. Rev. Entomol..

[57] Godfray, H. C. J. *Parasitoids: behavioral and evolutionary ecology*. (Princeton University Press, 1994).

[58] Duputie A, Rutschmann A, Ronce O, Chuine I (2015). Phenological plasticity will not help all species adapt to climate change. Glob. Change Biol.

[59] Van Nouhuys S, Lei G (2004). Parasitoid–host metapopulation dynamics: the causes and consequences of phenological asynchrony. J. Anim. Ecol..

[60] Benrey B, Denno RF (1997). The slow-growth-high-mortality hypothesis: a test using the cabbage butterfly. Ecology.

[61] Uesugi A (2015). The slow-growth high-mortality hypothesis: direct experimental support in a leafmining fly. Ecol. Entomol..

