# Supplementary material for: Differential responses of body growth to artificial warming between parasitoids and hosts and the consequences for plant seed damage
Source: Sci Rep. 2017 Nov 13;7:15472. doi: 10.1038/s41598-017-15453-y (PMC5684347; doi:10.1038/s41598-017-15453-y)

Differential responses of body growth to artificial warming between parasitoids and hosts and the consequences for plant seed damage

Xinqiang Xi[1], Yangheshan Yang[1], Xiaocheng Yang[2], Sören Nylin[3], Nico Eisenhauer[4,5], Shucun Sun[1,6,*]

[1] Department of Ecology, School of Life Science, Nanjing University, 163 Xianlin Avenue, Nanjing 210023, China

[2] College of Materials and Chemistry & Chemical Engineering, Chengdu University of Technology, East 3 Road ErXian Bridge ChengHua District, Chengdu 610059, China

[3] Department of Zoology, Stockholm University, SE-106 91 Stockholm, Sweden

[4] German Centre for Integrative Biodiversity Research (iDiv) Halle-Jena-Leipzig, Deutscher Platz 5e, 04103 Leipzig, Germany

[5] Institute of Biology, Leipzig University, Deutscher Platz 5e, 04103 Leipzig, Germany

[6] Center for Ecological Studies, Chengdu Institute of Biology, Chinese Academy of Sciences, 9 Section 4, Renminnan Rd, Chengdu 610041, China

[*] Corresponding author, email: shcs@nju.edu.cn

Figure S1. Temperature variation in warming and ambient treatments. Flowering periods are indicated by double headed arrows for each host plant species.

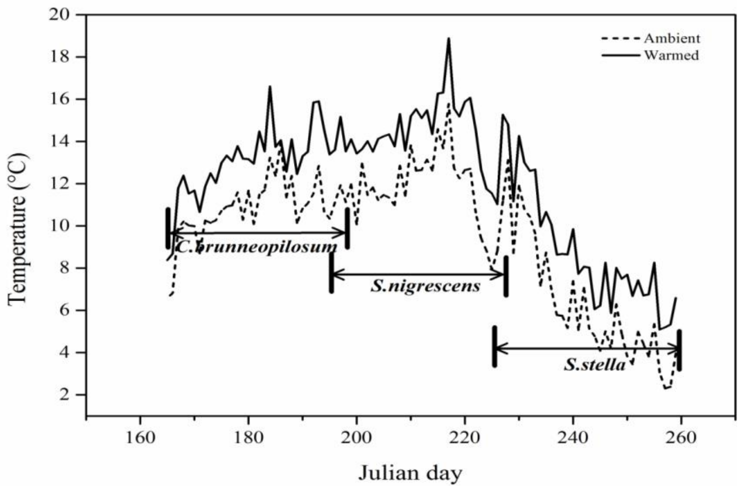

Figure S2. Total number of seeds per capitulum for three host plants (a, *Cremanthodium brunneopilosum*; b, *Saussurea nigrescens*; and c, *Saussurea stella*) in four different treatments (unparasitized and parasitized maggots in ambient and warmed plots).

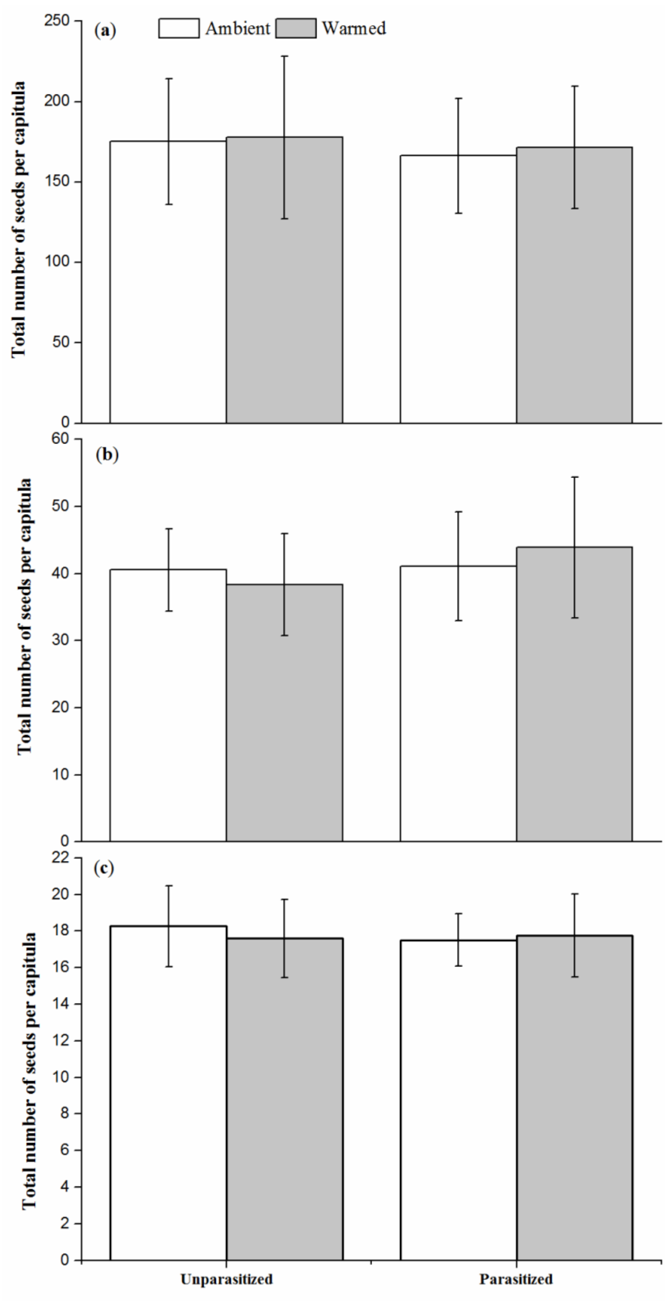

Figure S3. Individual seed mass for three host plants (a, *Cremanthodium brunneopilosum*; b, *Saussurea nigrescens*; and c, *Saussurea stella*) in four different treatments (unparasitized and parasitized maggots in ambient and warmed plots).

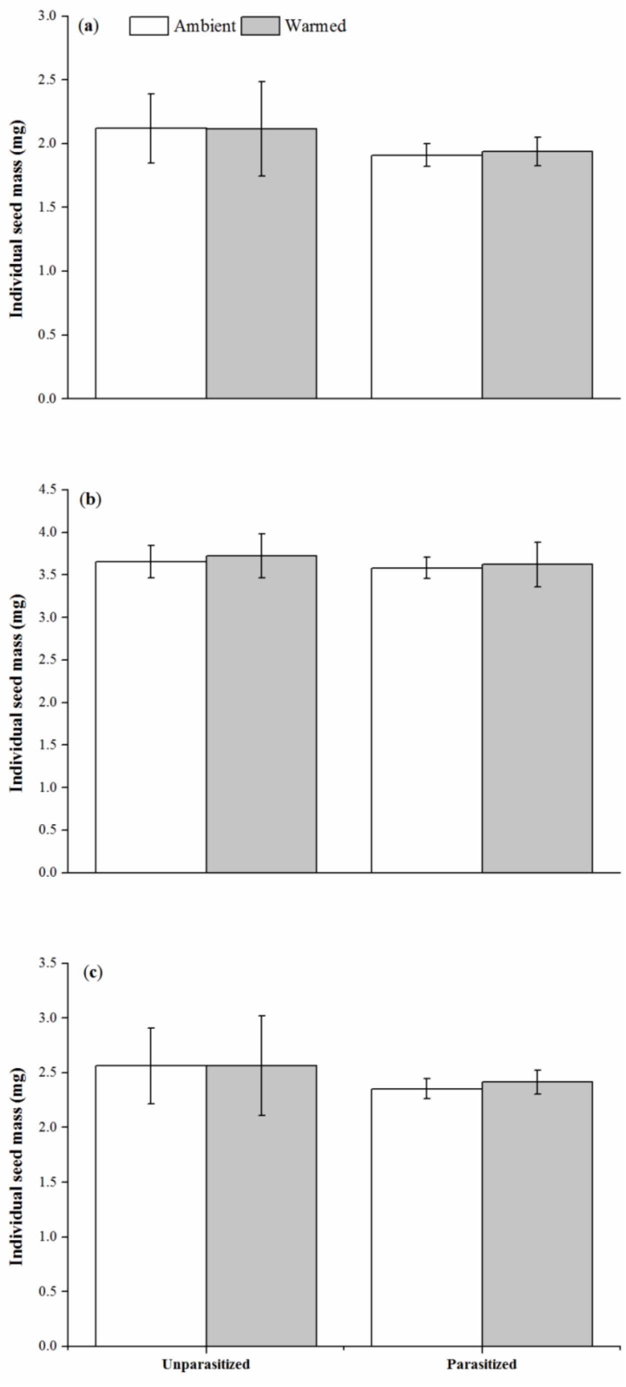

Supplement: Supplementary file 1 — Supplementary material [file 41598_2017_15453_MOESM1_ESM.pdf]
